# Fate of Tableted Freeze-Dried siRNA Lipoplexes in Gastrointestinal Environment

**DOI:** 10.3390/pharmaceutics13111807

**Published:** 2021-10-28

**Authors:** Asad Ur Rehman, Virginie Busignies, Marcela Coelho Silva Ribeiro, Nayara Almeida Lage, Pierre Tchoreloff, Virginie Escriou, Christine Charrueau

**Affiliations:** 1 Université de Paris, CNRS, INSERM, UTCBS, F-75006 Paris, France; asad-ur.rehman@u-paris.fr (A.U.R.); marcelacsribeiro@gmail.com (M.C.S.R.); nayara.almeidalage@gmail.com (N.A.L.); virginie.escriou@u-paris.fr (V.E.); 2 Univ. Bordeaux, CNRS, Arts et Metiers Institute of Technology, Bordeaux INP, INRAE, I2M Bordeaux, F-33400 Talence, France; virginie.busignies-goddin@u-bordeaux.fr (V.B.); pierre.tchoreloff@u-bordeaux.fr (P.T.); 3 Faculdade de Farmácia, Universidade Federal de Minas Gerais, Belo Horizonte 31270-901, MG, Brazil

**Keywords:** oral delivery, RNA interference, solid dosage form, gel electrophoresis, nanocarriers

## Abstract

The incorporation of siRNA into nanocarriers is mandatory to facilitate its intracellular delivery, as siRNA itself cannot enter cells. However, the incorporation of these nanocarriers into oral, solid dosage forms and their fate in the gastrointestinal environment is yet to be explored. In the present work, the fate of, (i) naked siRNA, (ii) freshly prepared siRNA lipoplexes, and (iii) tableted siRNA lipoplexes, in simulated gastric and intestinal fluids was studied. The siRNA, either released from or protected within the lipoplexes, was quantified by gel electrophoresis and siRNA efficacy was assessed in cell transfection. The freshly prepared lipoplexes kept their siRNA load and transfection efficiency totally preserved during 1 h of incubation in simulated gastric fluid at 37 °C. However, in simulated intestinal fluid, despite no release of siRNA from lipoplexes after 6 h of incubation, gene silencing efficacy was dramatically decreased even after 1 h of exposure. The lipoplexes obtained from tablets efficiently protected siRNA in simulated gastric fluid, thus preserving the gene silencing efficacy, whereas their incubation in simulated intestinal fluid resulted in a marked siRNA release and decreased gene silencing efficacy. These results provided a detailed explanation for understanding the fate of siRNA in gastrointestinal conditions, when simply loaded in lipoplexes or formulated in the form of tablets.

## 1. Introduction

Therapeutic RNA interference (RNAi) relies on the principle of silencing disease-related genes in a sequence-specific manner. After two decades of research, this promising curative modality has become a reality, since three siRNA therapeutics have been approved, i.e., ONPATTRO^®^ (patisiran, Sanofi Genzyme) in 2018 [1], GIVLAARI^®^ (givosiran, Alnylam Pharmaceuticals) in 2019 [2], both being commercialized, and finally OXLUMO^®^ (lumasiran, Alnylam Pharmaceuticals) in 2020 [3]. In ONPATTRO^®^, siRNA is formulated in lipid nanoparticles for the intravenous treatment of hereditary amyloidogenic transthyrethin amyloidosis with polyneuropathy in adults [2]. GIVLAARI^®,^ an aminolevulinate synthase 1 (ALAS1)-directed siRNA used to treat acute hepatic porphyria in adults [4], and OXLUMO^®^, an siRNA targeting the mRNA for the hydroxyacid oxidase 1 gene to treat primary hyperoxaluria type 1 [3], are both covalently linked to a ligand enabling their specific delivery to hepatocytes (Gal-NAc-siRNA conjugates), and are administered by subcutaneous injection.

The administration of nonconjugated siRNAs necessitates their formulation into vectors in order to achieve cellular delivery, since these negatively charged and water-soluble molecules, with high molecular weight and very low membrane permeability, are unable to cross cell membranes on their own. Currently, the administration route is essentially parenteral, but the oral route of delivery may be considered according to its advantages over the injectable route, i.e., ease of administration, non-invasive and painless for the patient, higher compliance, reduced health-care cost, and possibility to cure local and systemic diseases [5]. Generally, oral delivery of drugs represents a real challenge since physiological and cellular barriers have to be overcome. The physiological barriers include (i) the pH variations along the gastrointestinal tract, ranging from harsh acidic environment in the stomach to 5–9 pH values within the intestine, (ii) the presence of bile salts and degradative enzymes such as pepsin, amylase, trypsin, lipase, (iii) fluid flow and peristaltic activity, (iv) glycocalyx and mucus layers. The cellular barriers include the intestinal mucosa (comprising three layers, i.e., epithelium, lamina propria, and muscularis mucosa), the lymphoid follicles and Peyer patches. Specifically speaking of siRNAs, under their naked form, they would theoretically not be altered at acidic pH, nor be sensitive to proteolytic enzymes such pepsin in the stomach and trypsin in the intestine, nor to glycoside hydrolases such as amylase. However, siRNAs may be degraded by endonucleases within the gastrointestinal lumen [6,7,8]. As for lipid nanoparticles of siRNA, they could be altered by lipases, or retained by the mucus. Finally, the cellular barriers of the intestinal mucosa could represent either an obstacle to the absorption of siRNA vectors intended for a systemic effect, or a target for the local treatment of gastrointestinal diseases [9].

Although challenging, the oral route represents an emerging route for the administration of siRNAs, and their fate in gastrointestinal conditions has been explored, either in vitro with phosphate buffers at pH 2–4.5–7 [10], or with simulated gastric fluid SGF and simulated intestinal fluid SIF [11,12,13,14], or ex vivo with gastric and intestinal fluids harvested from rodents [15,16,17], showing that siRNAs formulated into nanoparticles (NPs) could resist, to some extent, the gastrointestinal challenges. In addition, considering administered *per os* in preclinical studies, some of these siRNA NPs allowed small intestine and colon epithelial cell entry [14], or systemic translocation into liver, spleen, lung or kidney [11,15], and even distant biological effects in liver [15,16,17,18]. For in vivo studies, the siRNAs NPs were administered either in liquid colloidal suspensions, or loaded in microspheres (reviewed in [5,19]), or encapsulated in exosomes [10]. These formulations were administered by gavage in rodents.

Beside these forms, our team recently patented an innovative dosage form consisting of tablets containing freeze-dried siRNA lipoplexes [20] that retained the bilayer lipid structures of the particles as well as their gene silencing efficacy upon the freeze-drying and tableting processes [21], thus opening promising perspectives to oral administration of siRNA-based medicines. However, there is to date no data available regarding the stability of freeze-dried and tableted siRNA lipoplexes in gastrointestinal conditions, which is a prerequisite to their efficacy in vivo.

Therefore, the objectives of the present work were to determine the stability of naked siRNA and siRNA lipoplex regarding the physiological challenges of pH and enzymes—apart from any cellular barrier—in vitro in simulated gastric and intestinal fluids. Preliminarily, the effect of the pH value, at the step of preparation of the lipoplexes, was evaluated on their size and transfection efficacy in order to set up this parameter. Then, after exposure to gastrointestinal conditions, gel electrophoresis was implemented, siRNA either released from lipoplexes or protected within the lipoplexes was quantified, and siRNA efficacy was assessed in cell transfection. This study was carried out in parallel with naked siRNA in solution, freshly prepared siRNA lipoplexes in colloidal suspension, and tableted freeze-dried siRNA lipoplexes after resuspension, with the perspective to identify whether this new tableted dosage form would require further protection for improving oral delivery of siRNA.

## 2. Materials and Methods

### 2.1. Materials

The siRNA (unmodified) specific to luciferase (sense strand, 5′ CUU ACG CUG AGU ACU UCG AdTdT 3′) and nonsilencing siRNA used as a negative control (sense strand, 5′ UUC UCC GAA CGU GUC ACG UdTdT 3′) were obtained from Eurogentec (Seraing, Belgium). Sodium alginate (reference 180947), dry methanol (methanol 99.9%, extra dry, AcroSeal), glucose, sucrose, pepsin (reference P7000), and pancreatin (reference P7545) were purchased from Sigma-Aldrich (St. Quentin Fallavier, France). The cationic lipid (2-{3-[bis-(3-amino-propyl)-amino]-propyl amino}-N-ditetradecyl carbamoyl methyl-acetamide) (DMAPAP) was synthesized in the laboratory as described [22]. The zwitterionic lipid (1,2-dioleoyl-sn-glycero-3-phosphoethanolamine) (DOPE) was purchased from Avanti Polar Lipids (Coger SAS, Paris, France), trehalose (Treha^®^ 16400) from Cargill (Paris La Défense, France), mannitol (Pearlitol^®^ 400 DC) from Roquette (Lestrem, France), magnesium stearate from Cooper (Melun, France), lactose (Flowlac^®^ 90) from Meggle (Wasserburg, Germany) and polyacrylamide gel (Novex^®^ TBE-Urea gel 6%) and TBE-Urea sample buffer 2x were obtained from Invitrogen (Paris, France).

### 2.2. Lipoplex Preparation

Firstly, the cationic liposomes were prepared by using the lipid film method with the cationic lipid DMAPAP and a zwiterrionic lipid DOPE (1/1, M/M) as previously reported by our research group [21,23,24]. As a brief description, the lipids were dissolved in chloroform (in equimolar amounts) followed by evaporation of the organic solvent resulting in the formation of a thin lipid film. The dried lipid film was rehydrated with MilliQ water, then a homogenous suspension of liposomes at 20 mM was obtained by extrusion of the suspension using the miniextruder (Avanti Polar Lipids) by carrying out 20 passages through a 0.4 µm-porosity polycarbonate membrane.

**Figure 1:**
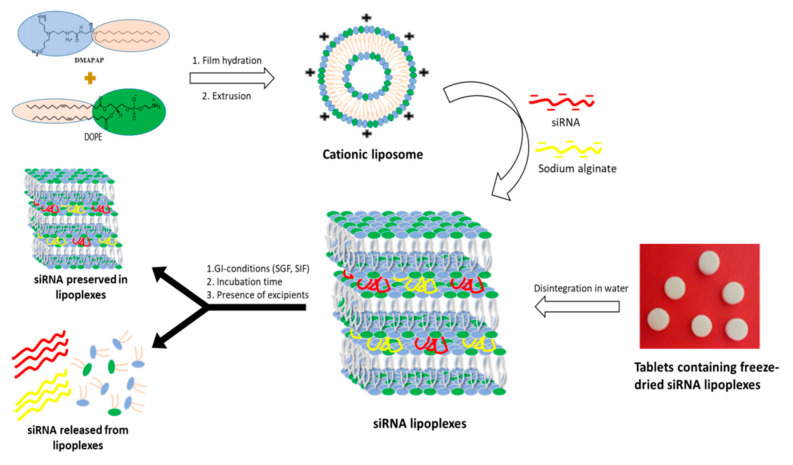
Preparation of siRNA lipoplexes and evaluation of the stability of siRNA and siRNA lipoplexes in gastrointestinal (GI) conditions.

Lipoplexes were prepared by mixing a specified volume of this liposomal suspension (diluted in 150 mM NaCl) with an equal volume of a solution containing siRNA (luciferase or control) and sodium alginate (ratio 1/1, *w*/*w*) in 150 mM NaCl (Figure 1). This mixture was allowed to mature into lipoplexes over a period of 1 h (at room temperature) and then the resulting lipoplexes characterized by a condensed multilamellar structure observed in transmission electron microscopy (TEM, see Figure 3 in ref. [25]) were used for further studies. The ± charge ratio (±R = 8) was calculated using the molar ratio of positive charges (3 positive charges per molecule of DMAPAP) and the molar ratio of negative charges (3.03 nmol and 5.05 nmol of negative charges/mg of siRNA and sodium alginate, respectively).

The effect of pH (2, 4.5 and 7) on the formation, stability and gene-silencing efficacy of lipoplexes was assayed in order to determine the best procedure for preparing stable and efficient lipoplexes. For that purpose, the size of the lipoplexes was measured for freshly prepared samples, and after 4 and 24 h of storage at room temperature.

### 2.3. Lipoplex Size Measurement

Size measurements were performed on a MALVERN Zetasizer Nanoseries Nano ZS using Malvern Zetasizer Software (medium: water, refractive index: 1.330, viscosity: 0.8872 cP, wavelength: 633 nm, refractive index of material polystyrene latex: 1.590, dielectric constant: 80.4). Particle suspensions were prepared in 100 μL of NaCl 150 mM with a final concentration of siRNA of 334 nM.

### 2.4. Lipoplex Freeze-Drying and Tableting of the Freeze-Dried Lipoplexes

The obtained lipoplexes were freeze-dried as described in our previous study [21]. Briefly, aqueous excipient solution (2.5% *w/v* of trehalose with 1% *w/v* of mannitol) was added to the lipoplex suspension (9.4 μM siRNA). The resulting suspension (4 mL) was taken in a 50 mL Falcon centrifuge tube (Corning, New York, NY, USA) and frozen in liquid nitrogen for 10 min and then it was connected to the drying chamber of a laboratory-scale freeze dryer (Christ-α 1-4, Christ, Osterode am Harz, Germany). A 30 h freeze drying cycle was performed and the process parameters were kept constant during the runs (the condenser chamber temperature was −60 °C and the chamber pressure was 0.1 mbar).

The freeze-dried solids (76.5% *w/w*) were mixed with Flowlac^®^ (23% *w/w*), and magnesium stearate (0.5% *w/w*) to prepare 160 mg of the final mixture. Then, the mixture was compacted with a StylOne Evolution compression simulator (Medelpharm, Beynost, France), and tooled with bevel-edged Euro B punches (6 mm in diameter). Compaction pressure of 150 MPa was applied using the force-driven mode of Analis software. Various characteristics of the formed tablets, such as apparent densities, thickness, diameter, elastic recovery, and cohesion have already been studied and reported by our group [21]. The scanning electron microscopic (SEM, see Figure 4 in ref. [21]) analysis and the elastic recovery (% ERT) measurements were also performed and reported [21]. For further studies, one tablet was disintegrated in 1 mL milliQ water to prepare suspension of lipoplexes.

### 2.5. Disintegration Test

The disintegration time of tablets was determined according to the European Pharmacopoeia 10.0, using test A for tablets and capsules of normal size, described in 2.9.1. “Disintegration of tablets and capsules” [26]. Briefly, one tablet was placed in each of the six tubes and the basket rack assembly was operated under vertical motion in one liter of water maintained at 37 °C until disintegration of the tablets. The assay was repeated in simulated gastric fluid as well.

### 2.6. Evaluation of the Stability of siRNA and Lipoplex es in Gastrointestinal (GI) Conditions

The protocol for testing the stability of siRNA and lipoplexes in gastrointestinal conditions took into account the parameters to which a pharmaceutical form would be exposed within the gastrointestinal tract after oral administration, i.e., the physiological temperature of 37 °C, the pH variations along the tractus, and the transit time according to the recommendations on dissolution testing by the European Pharmacopoeia [27] (Figure 1).

#### 2.6.1. Effect of Simulated Gastric Conditions on the Stability of siRNA and Lipoplexes

The naked siRNA (3.6 µM), freshly prepared siRNA lipoplexes (containing 3.6 µM siRNA) as well as the lipoplexes resuspended by disintegrating a tablet (containing freeze-dried siRNA lipoplexes 3.6 µM) in water, were incubated separately in SGF (34.2 mM NaCl, 3.2 mg/mL pepsin, pH 1.2 adjusted with HCl) [26] at 37 °C for 1 h. In parallel, similar samples were incubated with NaCl (150 mM) at 37 °C for 1 h and a third series of samples was treated with NaCl (150 mM) without any incubation (nonincubated samples), including the naked siRNA as control sample. At the end of the incubation, the lipoplexes were dissociated, in half of the samples, by the addition of NaCl (58.5 mg/mL) + Triton X-100 (200 mg/mL) mixture and remaining lipoplex samples were left untreated. The siRNA released or preserved within the lipoplexes was quantified by gel electrophoresis (followed by imaging).

#### 2.6.2. Effect of Simulated Intestinal Conditions on the Stability of siRNA and Lipoplexes

A similar set of samples was prepared and same protocol was followed, as in the case of SGF, however, the samples were incubated for 1 h, 3 h and 6 h in SIF (25mM KH_2_PO_4_, 10 mg/mL pancreatin, pH adjusted to 6.8 with NaOH) at 37 °C [27].

The effects of the excipients used in the preparation of the tablets on the stability of the siRNA and lipoplexes were also studied by using a placebo tablet (i.e., without freeze- dried lipoplexes). The placebo tablet was disintegrated in water. The resulting preparation was mixed with fresh lipoplexes and incubated in SGF and SIF at 37 °C. The siRNA released or preserved within the lipoplexes was quantified by gel electrophoresis.

### 2.7. Quantification of siRNA Released from or Preserved within Lipoplexes by Gel Electrophoresis

**Figure 2:**
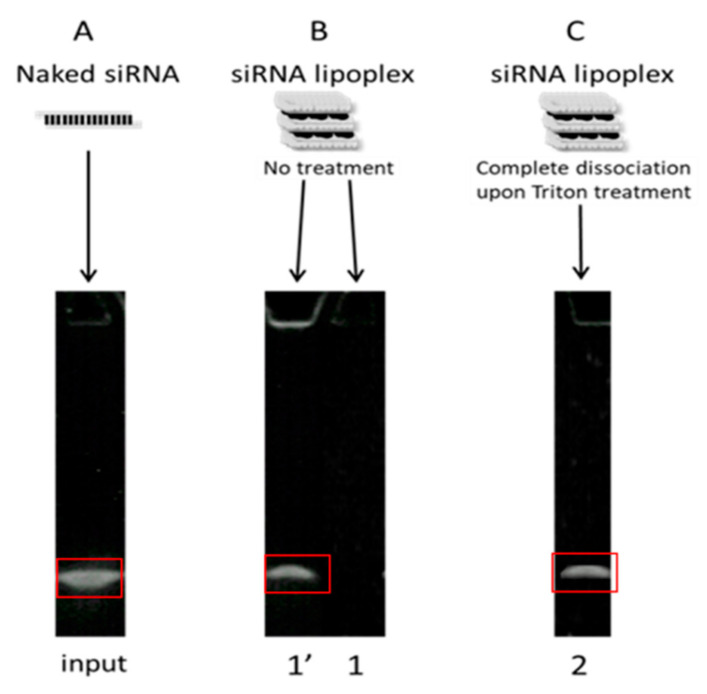
Diagram of siRNA integrity analysis on acrylamide gel. (**A**) When analyzed by gel electrophoresis, intact, naked, double-stranded siRNA appeared as a band. (**B**) siRNA lipoplexes were loaded on acrylamide gels, (1) if all the siRNAs were complexed in the assayed lipoplexes, no band was detected, (1′) whereas a band was detected if some of the siRNA payload leaked out. (**C**) Treatment of siRNA lipoplexes with Triton X-100 completely dissociated particles, releasing their siRNA payload (2). Band intensity (from the intact band) was then quantified with Image J. A red rectangular selection area was selected to measure the raw integrated density of each band. Results were expressed relative to naked siRNA.

The NaCl/Triton-treated as well as untreated lipoplexes were taken, and TBE-urea sample buffer 2× was added into each sample. These samples were then electrophoresed on 6% polyacrylamide gels in TBE (Tris base 89 mM, orthoboric acid 89 mM, EDTA 2mM, pH 8.3) running buffer at 180 V for 40 min. The gels were recovered and put into a SYBR^®^ Green II (Invitrogen, Paris, France) bath for 20 min and the stained siRNA bands were visualized on an ultraviolet (UV) transilluminator, and the obtained images were processed by ImageJ^®^ (Image Processing and Analysis in Java, open source software, original ImageJ 1.x version) to quantify siRNA. The naked siRNA band was used as a control (i.e., 100%) for the quantification of other bands on the gel. The % of preserved siRNA (within lipoplexes) and the % of released siRNA were obtained as described in Figure 2. Results were expressed relative to naked siRNA (designated hereafter as “input”). Relative band intensities (I) were derived by using Image J to calculate integrated optical density of each band. First of all, a rectangular area was created around the control/naked siRNA band on the image of the gel, and raw integrated density, i.e., the sum of all the pixel intensities, was measured for each condition without modifying the size of the selection (Figure 2). To cancel the effect of background in the measurements, the raw integrated density of the background was also measured by the same procedure and this value was subtracted from the raw integrated density of each band. The amount of intact, released siRNA was given by (1′), the amount of intact siRNA remaining in lipoplexes was given by (2)−(1′) (Figure 2).

The % of intact siRNA preserved in lipoplexes was then given by: 100 × [I(band 2) − I(band 1′)]/I(input), while the % of released intact siRNA was given by: 100 × I(band 1′)/I(input), as previously described in Hamoudi et al. [28].

### 2.8. Evaluation of Gene Silencing Efficacy by Cell Transfection

B16-Luc were obtained and transfected as described [24,25]. Lipoplex suspensions (freshly prepared or resuspended from tablets) were diluted, as such or after incubation with SIF or SGF for 1 h at 37 °C, in complete culture cell medium (siRNA concentration 37.5 nM) and added to cells in triplicate for 24 h in 24-well plates. Transfection medium was replaced by fresh medium for another 24 h, then the transfected cells were washed twice with PBS and lysed, and luciferase and total protein were assayed as in [24]. The results, calculated in cps (count per second), were normalized to the total protein concentration of each sample, and the gene-silencing efficacy was expressed as the luciferase activity inhibition percentage relative to the luciferase activity of nontransfected control cells.

### 2.9. Statistical Analysis

All results were analyzed with the unpaired, nonparametric Mann−Whitney test using two-tailed P-values. Results with *p* < 0.05 were considered statistically significant.

## 3. Results

Lipoplexes were prepared by mixing a DMAPAP and DOPE liposome suspension with siRNA, sodium alginate and NaCl in aqueous solution as previously reported by our research group [21,23,24]. They were tested as freshly prepared siRNA lipoplexes in colloidal suspension, and tableted, freeze-dried siRNA lipoplexes after resuspension, in comparison with naked siRNA in solution (Figure 1).

### 3.1. Disintegration Test

The uncoated tablets must disintegrate within 15 min to meet the standards of United States pharmacopoeia (USP) and European pharmacopoeia (EP). In this case, all six tablets that were obtained under a compression pressure of 150 MPa were disintegrated within five min, 4 min 54 s and 4 min 46 s precisely in water and SGF, respectively. Therefore, the tablets containing freeze-dried siRNA meet the standards of the USP and EP.

### 3.2. Effect of pH on Formation, Stability, and Gene Silencing Efficacy of Lipoplexes

Oral administration presents a number of limitations, one of which is the acidic-to-near-neutral pH of the gastrointestinal tract. We first assessed the impact of preparing and incubating siRNA lipoplexes in acidic pH medium on the size of lipoplexes and efficacy of gene silencing.

The lipoplexes were prepared at different pH levels, namely 2, 4.5 or 7, and their average size was measured immediately after preparation and after 4 h and 24 h of storage at room temperature following their preparation as well.

**Figure 3:**
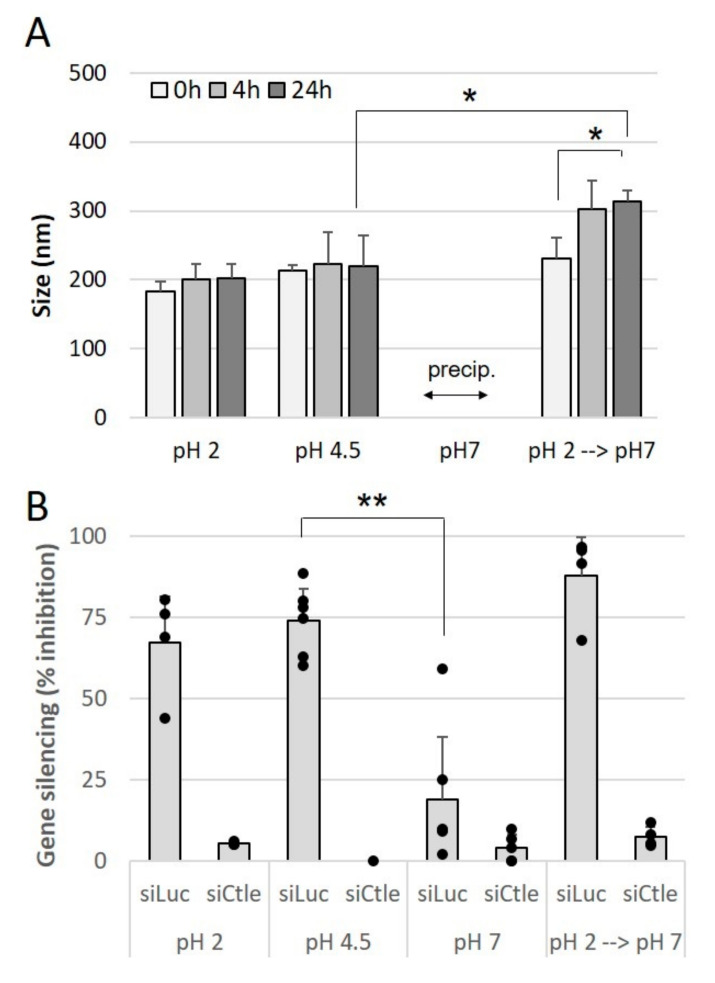
Effect of pH on size and gene-silencing efficacy of siRNA lipoplexes. siRNA lipoplexes were prepared at pH 2, 4.5 or 7, or prepared at pH 2 and incubated for 1 h at pH 7. **A**—Size measurements were performed immediately after preparation, and 4 h and 24 h after preparation. **B**—siRNA lipoplexes were prepared with siRNA anti-luciferase (siLuc) or siRNA control (siCtle) and applied on B16-Luc at the same final concentration of siRNA (37.5 nM). The results were expressed as % inhibition of luciferase activity relative to nontransfected cells (means ± SD, *n* = 3). * *p* < 0.05, ** *p* < 0.01.

Figure 3A shows that the lipoplexes prepared at acidic pH, 2 or 4.5, exhibited a size of around 200 nm which remained stable for 24 h. On the other hand, at pH 7, the lipoplexes precipitated immediately after their preparation. When they were prepared at acidic pH and brought to neutral pH, an increase in size, from 200 nm to 300 nm, was observed and these lipoplexes remained stable afterwards. Then, the impact of pH on the gene silencing efficacy of lipoplexes prepared at different pH was evaluated. As shown in Figure 3B, lipoplexes prepared at acidic pH, 2 or 4.5, exhibited a gene silencing efficacy of around 70%, while lipoplexes prepared at neutral pH only exhibited 20% efficacy. In addition, lipoplexes prepared at pH 2 and passed to neutral pH retained their efficacy. These results show that the most-effective lipoplexes are formed at acidic pH, and these effective lipoplexes, once prepared, retain their efficacy when they are subjected to pH variations.

### 3.3. Evaluation of the Stability of siRNA and Lipoplexes in Gastrointestinal (GI) Conditions

#### 3.3.1. Effect of Simulated Gastric Conditions on the Stability of Naked siRNA and siRNA Lipoplexes

**Figure 4:**
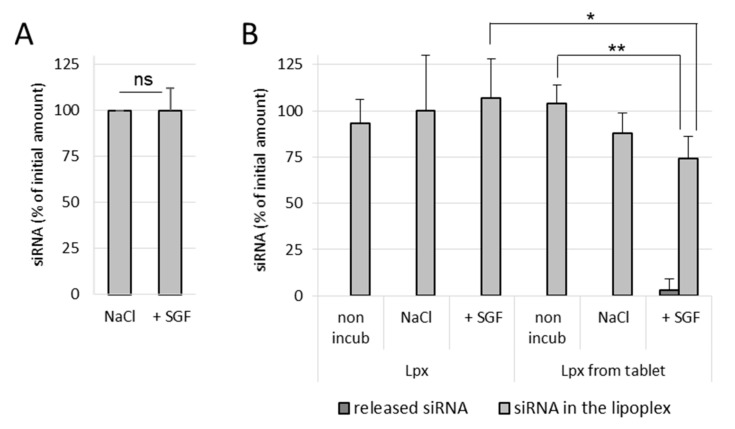
Effect of NaCl (150mM) and simulated gastric conditions on the stability of naked siRNA (**A**) and siRNA lipoplex (**B**). (**A**) Naked siRNA was incubated in NaCl or SGF for 1 h at 37 °C, before being assayed by gel electrophoresis. (**B**) siRNA lipoplexes freshly prepared (Lpx) or resuspended from tablets (Lpx from tablet) were incubated for 1 h, at 37 °C in NaCl or SGF. Nonincubated (non-incub) samples were also studied. The percentage of siRNA released or preserved within lipoplex was quantified by gel electrophoresis as described in Material and Methods. No siRNA release was observed in the case of freshly prepared lipoplexes as well as lipoplexes resuspended from tablets, either nonincubated or incubated in NaCl. The results were expressed as % of initial amount of siRNA used (mean values ± SD, A *n* = 3; B *n* = 5; * *p* < 0.05, ** *p* < 0.01, ns: not significant).

It was observed that naked siRNA remained stable in NaCl as well as in SGF, even after 1 h incubation at 37 °C, which indicates that the gastric conditions do not degrade naked siRNA (Figure 4A). In NaCl (150 mM)-treated lipoplex samples, no release of siRNA was observed in both incubated as well as nonincubated samples, which shows that the siRNA lipoplexes (fresh lipoplexes and the ones resuspended from tablets) remain stable in NaCl (Figure 4B). Similarly, fresh siRNA lipoplexes (in suspension) also did not show any release of siRNA after 1 h incubation in SGF with complete preservation of siRNA in lipoplexes. However, in the tableted, freeze-dried siRNA lipoplex (after disintegration of the tablet) samples, a low release of siRNA was obtained after 1 h incubation in SGF with ~74% intact siRNA preserved in lipoplexes. This shows that the siRNA is released to some extent (after 1 h incubation) from the tableted, freeze-dried lipoplexes, but the majority of siRNA content is well preserved in the tablets (Figure 4B). Thus, it can be concluded that the siRNA and the lipoplexes (fresh as well as tableted) are rather resistant to the gastric conditions despite the pressure constraint applied on tableted siRNA lipoplexes. However, it is not possible to know whether the greater fragility of the lipoplexes resuspended from tablet, shown by the decrease in the percentage of siRNA preserved in the lipoplexes (obtained from the tableted versus the suspension sample), is due to the gastric medium or the presence of excipients (Flowlac^®^ 37 mg/mL, magnesium stearate 0.8 mg/mL, trehalose 90 mg/mL, mannitol 36 mg/mL).

#### 3.3.2. Effect of Simulated Intestinal Conditions on the Stability of Naked siRNA and siRNA Lipoplexes

**Figure 5:**
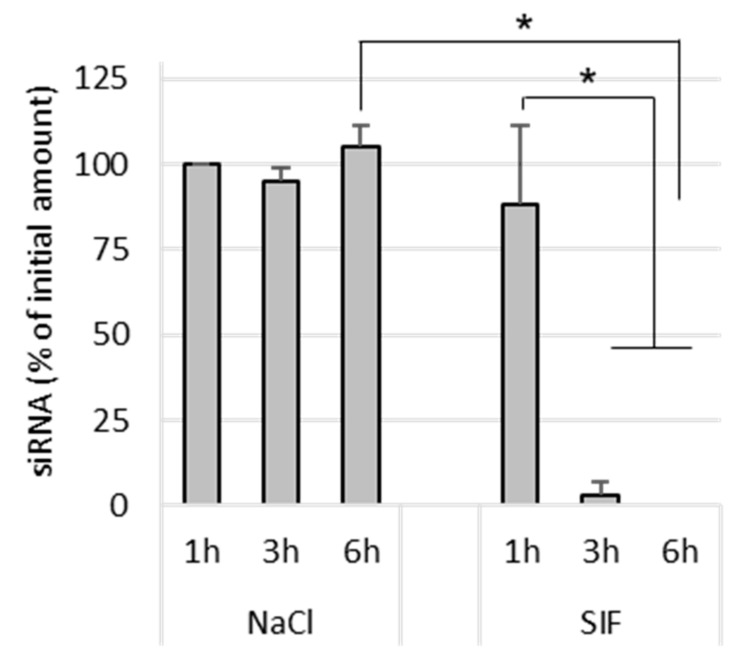
Effect of NaCl (150 mM) and simulated intestinal conditions on siRNA stability. Naked siRNA was incubated for 1 h, 3 h and 6 h at 37 °C in NaCl or SIF, before being assayed by gel electrophoresis. The results are expressed as % initial amount of siRNA used (mean values ± SD, *n* = 3; * *p* < 0.05).

Naked siRNA remained stable in NaCl after 6 h incubation at 37 °C. In SIF-treated samples, naked siRNA was completely degraded after 3 h incubation (Figure 5). This shows that the siRNA is degraded by its exposure to the intestinal environment, probably by enzymes.

**Figure 6:**
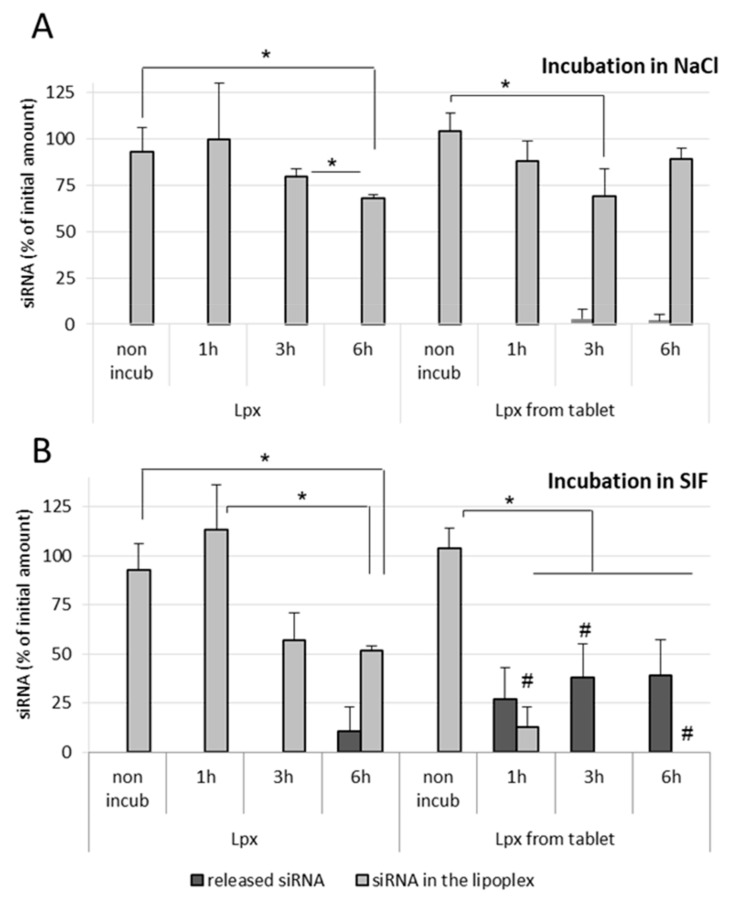
Effect of NaCl (150 mM) (**A**) and simulated intestinal conditions (**B**) on the stability of siRNA lipoplexes. siRNA lipoplexes freshly prepared (Lpx) or resuspended from tablets (Lpx from tablet) were incubated for 1 h, 3 h and 6 h at 37 °C in NaCl (**A**) or SIF (**B**). Nonincubated (non-incub) samples were also studied. The percentage of siRNA released or preserved within lipoplex was quantified by gel electrophoresis as described in Material and Methods. The results were expressed as % initial amount of siRNA used (mean values ± SD, *n* = 3–5; * statistical significance between different treatments applied to the same type of lipoplex, * *p* < 0.05; # statistical significance between Lpx and tableted Lpx undergoing the same treatment, # *p* < 0.05).

In the case of NaCl (150 mM)-treated fresh lipoplex samples, no release of siRNA was observed in both nonincubated as well as incubated samples, even after 6 h incubation at 37 °C. However, tableted, freeze-dried siRNA lipoplexes showed a very low release of siRNA after 3 h as well as after 6 h incubation (Figure 6A). In SIF-treated fresh lipoplex samples, no release of siRNA was observed after 1 h and 3 h incubation, however an almost 50% decrease in intact siRNA within lipoplexes was obtained after 3 h incubation in SIF. After 6 h incubation in SIF, a significant amount of siRNA (>10%) was released from the fresh lipoplexes with further decrease in the intact siRNA content within lipoplexes (Figure 6B). grotected by the lipoplexectrophoresiswas.

In the case of tableted lipoplexes, release of siRNA was observed after 1 h incubation in SIF (>25%), which kept increasing after 3 h and remained stable after 6 h incubation, respectively. The amount of intact siRNA present within the tableted lipoplexes was found around 13% after 1 h incubation in SIF, with no intact siRNA recovered from tableted lipoplexes after 3 h incubation (Figure 6B). It can be concluded that the fresh lipoplexes are more resistant towards the intestinal environment (enzymes) when compared with tableted lipoplexes.

#### 3.3.3. Effect of Excipients on the Stability of Naked siRNA and siRNA Lipoplexes

The percentage of siRNA released from the fresh lipoplexes and the tableted lipoplexes, once submitted to intestinal environment conditions, was found to be different.

**Figure 7:**
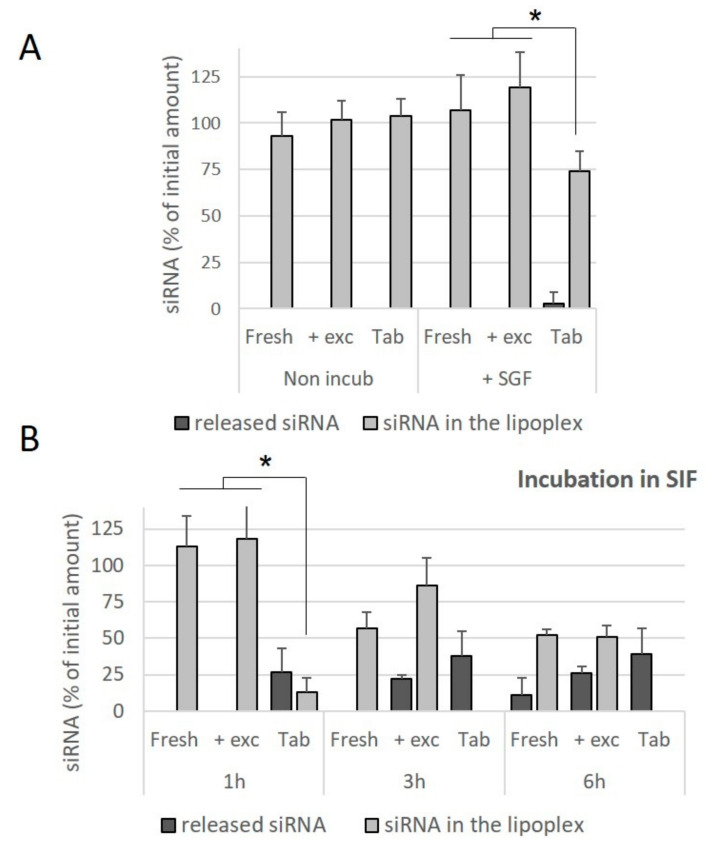
Effect of the incubation in excipients and simulated gastrointestinal conditions on the stability of siRNA lipoplexes. siRNA lipoplexes were diluted in excipients (trehalose, mannitol and lactose) obtained from the disintegration of a placebo (without lipoplexes) tablet (+ exc) and compared with freshly prepared lipoplex (Fresh) or lipoplex resuspended from tablets (Tab) after incubation for 1 h at 37 °C in SGF (**A**) or for 1 h, 3 h and 6 h at 37 °C in SIF (**B**). Nonincubated (Non-incub) samples were also studied. The percentage of siRNA released or preserved within lipoplex was quantified by gel electrophoresis as described in Material and Methods. The results were expressed as % initial amount of siRNA used (mean values ± SD, *n* = 3, * statistical significance between different treatments applied to the same type of lipoplex, * *p* < 0.05).

In order to assess whether this difference was due to the compressive stresses, or the presence of the excipients used for the preparation of the tablets, freshly prepared lipoplexes were incubated in an excipient solution and the effect of this incubation on the release of siRNA was evaluated. It was observed that the presence of excipients did not contribute towards the release of siRNA from the fresh lipoplexes incubated in SGF for 1 h (Figure 7A), nor towards a decrease in the amount of intact siRNA preserved within the lipoplex. Similarly, the presence of excipients did not contribute towards a release of siRNA from lipoplexes incubated in SIF for 1 h. At 3 h and 6 h of incubation in SIF, no significant effect of the presence of excipients was observed, whereas in these conditions of incubation, no intact siRNA is preserved within lipoplexes resuspended from tablets (Figure 7B). It can be concluded that tableted lipoplexes appeared to be weakened with respect to exposure to conditions in the intestinal environment.

### 3.4. Effect of Simulated Gastrointestinal Conditions on Transfection Efficacy

**Figure 8:**
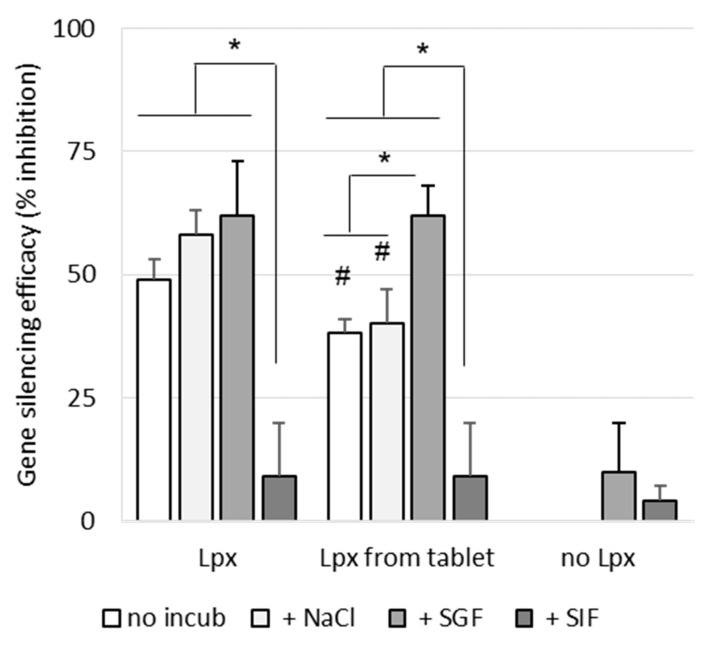
Effect of simulated gastrointestinal condition on gene-silencing efficacy of siRNA lipoplexes. siRNA lipoplexes freshly prepared (Lpx) or resuspended from tablets (Lpx from tablet) were incubated for 1 h at 37 °C in SGF, SIF or NaCl (150mM) or nonincubated (non-incub) before being applied on B16-Luc at the same final concentration of siRNA (37.5 nM). siRNA lipoplexes were prepared with siRNA anti-luciferase. The results were expressed as % inhibition of luciferase activity relative to nontransfected cells (means ± SD, *n* = 3; * *p* < 0.05; # statistical significance between Lpx and tableted Lpx undergoing the same treatment, # *p* < 0.05).

As shown in Figure 8, incubation of siRNA lipoplexes in NaCl (150 mM) or SGF for 1 h had no effect on their gene-silencing efficacy, while incubation in SIF induced an important loss of activity. As we have already shown [21], nonincubated lipoplexes resuspended from a tablet exhibited lower efficacy (by about 40%) than fresh lipoplexes. Incubation in saline induced no change in efficacy, while treatment with SGF even increased the observed efficacy. This increase could be due to the effect of highly acidic pH on luciferase activity, which was observed when SGF was applied to cells in the absence of lipoplexes.

As with fresh lipoplexes, incubation in SIF completely collapsed their efficacy. In summary, whether the lipoplexes were fresh or resuspended from a tablet, their efficacy was maintained after SGF challenge but a significant loss of activity was observed after SIF challenge.

## 4. Discussion

Oral delivery of siRNAs has been considered ever since the Nobel Prize was awarded to RNA interference in 2006 [6]. Hence, the first study reporting successful systemic delivery of siRNA after oral gavage of glucan-encapsulated siRNA microparticles was published in 2009 [29]. Since then, to our best knowledge, relatively few authors [10,11,12,13,14,15,16,17,18] have explored the capacity of siRNAs and their carriers to stand the challenging transit in the gastrointestinal tract and reach their intracellular target site intact and active, which is the prerequisite for efficient oral siRNA therapies. The stability of siRNAs in gastric and intestinal media can be considered at three levels: the naked siRNA molecule in solution, the siRNA loaded in a nanoparticulate vector in suspension, and the siRNA nanocarrier incorporated in a solid pharmaceutical oral dosage form.

In order to assess siRNA stability, we used an original method previously published by our research group that allowed quantification of both siRNA retained inside and released from its nanocarrier by assaying siRNA lipoplexes through gel electrophoresis without or with NaCl-Triton treatment, measuring integrated optical density for each siRNA band, and further calculating the percentages of intact siRNA either released in the medium or remaining in the lipoplex, relative to naked siRNA total load [28]. This method, completed with gene-silencing assays in cultured cells after siRNA (naked and formulated) exposure to simulated gastric and intestinal fluids, provided an informative view of siRNA’s fate in gastrointestinal conditions when simply loaded in lipoplexes or further incorporated in a tablet.

In our study, the naked siRNA in solution could resist gastric challenges for one hour at physiological temperature, but it was progressively altered in intestinal conditions, with a total degradation at 3 h. When documented in the literature, the stability of naked siRNA in gastric media varied according to the experimental conditions of the assays. Hence, siRNA tested in simulated gastric fluid at pH 1.2 without pepsin was stable for one hour at room temperature [11], while siRNA tested in gastric fluids from rodents was totally degraded within 20 min [15], or one hour at 37 °C [16,17,18]. If the temperature could explain partially these differences, the presence of nucleases in the fluids harvested from animals could also be involved in the degradation of naked siRNA at acidic pH. Indeed, Guo et al. have shown that naked siRNA, stable in simulated gastric fluid, was considerably degraded when extra nucleases were added into the medium [30]. The alteration of siRNA observed in our study in simulated intestinal fluid at pH 6.8 is in agreement with previously published data reporting a major degradation in simulated intestinal fluid within 30 min at 37 °C [30], and for a total degradation in intestinal fluids from rodents within 2 h at 37 °C [15,16,17,18]. This could be attributed to the presence of nucleases in the pancreatin extract added in the simulated intestinal fluid in our work and Guo’s study, and in the intestinal fluids collected in animals in the other studies. One single publication carried out at ambient temperature in simulated intestinal fluid without pancreatin showed indeed the stability of naked siRNA [11]. In summary, pH in itself, between 1 and 7, does not alter naked siRNA, but nucleases are deleterious at gastric and intestinal levels.

Since naked siRNA in itself cannot enter cells to exert RNA interference, its incorporation into nanovectors is mandatory to allow for its intracellular delivery. Several nanosystems of siRNA have been shown to protect, to some extent, siRNA from gastro-intestinal challenge, mainly including polymeric nanoparticles [11,15,16,17,18], but also cell-penetrating peptides–siRNA noncovalent nanocomplexes [12], amphiphilic polyallylamine-based polymeric micelles [30], lipidoid nanoparticles [14], and milk exosomes [10]. To our best knowledge, we report here for the first time the fate of polymeric–lipidic nanoparticles, namely lipoplexes, in gastrointestinal conditions. Interestingly, these nanoparticles were able to keep their siRNA load intact and unreleased for one hour in simulated gastric fluid at 37 °C; in addition, their transfection efficiency was totally preserved. However, in simulated intestinal fluid, despite the fact that no release of siRNA from lipoplexes could be detected before six hours of incubation, silencing efficacy was dramatically decreased after one hour exposure, showing that this medium was deleterious to siRNA lipoplex stability. This could be due to presence of bile salts in simulated intestinal fluid that could progressively destabilize the lipoplex structure and expose siRNA to degradative nucleases. In the same way, lipidoid nanoparticles could resist gastric challenge but they partially lost silencing efficacy when exposed to “fed” concentrations of pepsin and bile salts [14]. As a consequence, such nanocarriers should be protected from the intestinal environment in order to exert full therapeutic efficacy. This objective might be achieved through their inclusion into dosage forms devoted to oral administration, such as tablets and capsules.

Currently, the incorporation of siRNA nanovectors in solid oral dosage forms and further determination of their fate in gastrointestinal conditions are scarce. Indeed, to our best knowledge, one single article reported the formulation of cell-penetrating peptides–siRNA nanocomplexes into solid dispersions intended for oral delivery that are stable in simulated gastric conditions [12]. No compacted solid dosage forms such as tablets could be found in the literature as siRNA oral delivery forms. In our study, tablets obtained from freeze-dried lipoplexes were shown to efficiently protect siRNA in simulated gastric fluid—although a minimal release could be observed at one hour—allowing preservation of the majority of the gene-silencing efficacy. Exposure to simulated intestinal fluid for one hour also permitted preservation of about 13% of intact siRNA within the tableted lipoplexes. However, lipoplexes from tablets could not resist longer simulated intestinal fluid challenges, which resulted in a marked siRNA release and decreased gene-silencing efficacy, thus showing that the freeze-drying and tableting processes further weakened siRNA lipoplexes. Importantly, the tablets tested here were obtained under a compression pressure in the medium range of pressures previously tested between 50 and 250 MPa [21]. Amongst several advantages, tableted dosage forms can easily modulate the release kinetics of active ingredients through an adequate formulation. Two options might be considered in order to improve this promising oral dosage form and to bring tableted siRNAs to clinical applications. On the one hand, the lyophilization and compression parameters, for example, sample temperature during sublimation and lower compaction pressure, could be further optimized in order to reduce their impact on lipoplex stability. On the other hand, tablet formulation could be optimized by using excipients able to resist intestinal conditions, either under a matrix form, or as external film coatings.

## Data Availability

The data presented in this study are available in the present article.
